# Influence of Sub-Daily Variation on Multi-Fractal Detrended Fluctuation Analysis of Wind Speed Time Series

**DOI:** 10.1371/journal.pone.0146284

**Published:** 2016-01-07

**Authors:** Xianxun Wang, Yadong Mei, Weinan Li, Yanjun Kong, Xiangyu Cong

**Affiliations:** 1 State Key Laboratory of Water Resource and Hydropower Engineering Science, Wuhan University, Wuhan, Hubei, China; 2 Hubei Collaborative Innovation Center for Water Resources Security, Wuhan, Hubei, China; 3 POWERCHINA Kunming Engineering Corporation Limited, Kunming, Yunnan, China; East China University of Science and Technology, CHINA

## Abstract

Using multi-fractal detrended fluctuation analysis (MF-DFA), the scaling features of wind speed time series (WSTS) could be explored. In this paper, we discuss the influence of sub-daily variation, which is a natural feature of wind, in MF-DFA of WSTS. First, the choice of the lower bound of the segment length, a significant parameter of MF-DFA, was studied. The results of expanding the lower bound into sub-daily scope shows that an abrupt declination and discrepancy of scaling exponents is caused by the inability to keep the whole diel process of wind in one single segment. Additionally, the specific value, which is effected by the sub-daily feature of local meteo-climatic, might be different. Second, the intra-day temporal order of wind was shuffled to determine the impact of diel variation on scaling exponents of MF-DFA. The results illustrate that disregarding diel variation leads to errors in scaling. We propose that during the MF-DFA of WSTS, the segment length should be longer than 1 day and the diel variation of wind should be maintained to avoid abnormal phenomena and discrepancy in scaling exponents.

## Introduction

A renewable resource and potential energy source, wind power needs to be further explored in an effort to meet the increasing requirement for electricity and avoid depleting fossil resources, which are aggravating environmental pollution [1–2]. Wind is one kind of natural signal, and it is important to discover the regular patterns of wind. It has been found that there are long-range power-law correlations in wind speed time series (WSTS) [3]. These long-range power correlations could be used in wind feature research and the prediction of wind [4].

The auto-correlation function and power spectrum are traditional methods for capturing long-range power correlation. The spectrum *E*(*f*) follows a power law of the form *E*(*f*) ~ *f*^*-α*^ in log-log scale. Due to sensitivities to non-stationary effects, traditional methods are limited [3]. Recently, the detrended fluctuation analysis (DFA) [5] method has been widely applied for scaling analysis of non-stationary data. However, DFA is inadequate for addressing processes governed by more than one scaling exponent. Based on DFA, multi-fractal detrended fluctuation analysis (MF-DFA) [6] has been introduced and successfully applied in many fields [3–4, 7–20]. Many research studies have indicated that WSTS is multi-fractal and used MF-DFA to scale analysis of WSTS [3–4, 6–10].

In discussions of the effects of trends on scaling exponents in the literature, it was found that a crossover (i.e., inconsistency of scaling exponent) usually arises from a change in the correlation properties of the signal at different temporal or spatial scales or trends in the data [13]. As a natural signal, wind has features of diel variation and seasonal alternation. Some researchers have found inconsistencies in scaling exponents of WSTS from several days to seasonal time scales. The scaling exponent of WSTS is influenced by meteorology, climate, and weather patterns and displays different values in various ranges of segment length (*s*) [10–12]. Ref. [10] reveals that when *s* was larger than seasonal length, the scaling exponent became smaller. The author thought this might be linked to meteo-climatic phenomena. Ref. [11] notes that the change of the scaling exponent was caused by climate patterns, and in Ref. [12] it was due to alterations between dry and wet climates. The diel variation is another property of wind. With advances in anemometer equipment, a high resolution (i.e., < 1 h or 10 min) of WSTS can be observed. This means that sub-daily processes of wind can be contained in WSTS and the key parameter of MF-DFA, *s*, can be extended to a sub-daily scope. However, to our knowledge this expansion has not been attempted thus far. We assumed that due to differences in temporal scales there might be some inconsistencies in the scaling exponent if the range of *s* was extended to a sub-daily scale. With that hypothesis, we attempted to discuss the consequence of extending the lower range of *s* to sub-daily and the influence of diel variation on scaling analysis of WSTS.

## Materials and Methods

### Materials

In scaling analysis of wind, the material is a WSTS. The length of the WSTS refers to the actual length of time between the beginning and end of the WSTS, and the resolution of the WSTS refers to the length of time intervals of the WSTS. The WSTS analyzed in this research was provided by the Kunming Engineering Corporation Limited of POWER CHINA (Kunming, 650051). The sampling sites of this WSTS were located in the Yunnan province of southwest China. These locations are public regions in China, and no specific permissions were required. No endangered or protected species were involved. The resolution of this WSTS is 10 min. The details are listed in Table 1. The processes of those WSTSs could be found in S1–S10 Figs.

**Table 1:** Parameters of WSTS analyzed in this research.

Site No.	Longitude (E)	Latitude (N)	Height (m)	Starting date	Ending date
**1#**	103°0.811'	24°18.788'	2380	2011/09/06	2013/08/09
**2#**	102°28.645'	25°10.667'	2457	2008/09/01	2009/08/31
**3#**	102°26.242'	25°12.633'	2580	2008/05/14	2009/05/13
**4#**	101° 28.239'	25° 36.959'	2535	2011/07/19	2012/07/18
**5#**	101°4.657'	25°49.657'	2235	2013/02/01	2014/01/31
**6#**	101° 30.677'	25° 31.433'	2395	2011/12/11	2012/12/10
**7#**	101°28.729'	25°30.708'	2546	2011/12/11	2012/12/10
**8#**	101°25.949'	25°30.705'	2710	2011/12/11	2012/12/10
**9#**	101°25.545'	25°31.482'	2701	2011/12/11	2012/12/10
**10#**	101°24.327'	25°34.536'	2631	2011/10/16	2012/10/15

## Methods

### Multi-fractal detrended fluctuation analysis

Based on DFA, which was introduced by Peng et al. in 1994 [5], multi-fractal detrended fluctuation analysis (MF-DFA) was developed by Kantelhardt et al. in 2002 [6]. Both DFA and MF-DFA have been widely applied to fields including DNA sequencing [14], astronomy [15], pathology [16], economics [17], physics [18], hydrology [19], and aerography [20]. Podobnik and Stanley [21] generalized DFA and introduced dentrended cross-correlation analysis (DCCA) for bivariate time series, which led to the development of a group of multi-fractal DCCA methods (e.g., multi-fractal detrended cross-correlation analysis (MF-X-DFA) [22], multi-fractal detrending moving average cross-correlation analysis (MF-X-DMA) [23], multifractal height cross-correlation analysis (MF-HXA) [24], multi-fractal cross-correlation analysis based on partition function (MF-X-PF) [25], multi-fractal cross-correlation analysis (MF-CCA) [26], multi-fractal detrended partial cross-correlation analysis (MF-DPXA) [27]).

WSTS are univariate time series. When a WSTS is analyzed together with other variates, such as the air pollution index (API) [28], particulate matter 2.5 (PM2.5) [29], or solar radiation index [30], the bivariate methods mentioned above should be adopted. In most recent works, when WSTS are analyzed individually MF-DFA is applied to study the fluctuating feature wind speed [3–4, 7–12]. Ref. [7] earlier applied MF-DFA for a scaling analysis of a WSTS. DFA is appropriate for extracting the scaling exponent from mono-fractal signals, but is insufficient for multi-fractal signals that are characterized by more than one exponent [31]. MF-DFA, a generalization of DFA, is a robust and reliable method for capturing the scaling exponents of multi-fractal series [6]. In recent research, it has been noted that WSTS are multi-fractal [3–4, 7–12]. Detailed descriptions of MF-DFA can be found in Ref. [6]. Considering {*x*_*i*_}(*i* = 1, 2, …*N*) as a WSTS, the main steps of MF-DFA are as follows:

Step 1. Determine the “profile”.
  $$Y(j)=\sum_{i=1}^{j}\left(x_{i}-\bar{x}\right),(j=1,2,\ldots N)$$ (1)
  where *Y*(*j*) represents the profile series and $\bar{x}$ represents the average value of{*x*_*i*_}.
Step 2. Profile division.
  The profile series *Y*(*j*) is divided into several non-overlapping segments of equal length. Because the length of *N* of *Y*(*j*) is often not a multiple of the segment length *s*, a short tail may exist. To not lose any information from the profile, two divisions that start from the beginning and end are carried out. Thereby, 2*N*_*s*_(*N*_*s*_ = int(*N* / *s*)) segments are obtained.
Step 3. Local trend calculation and elimination.
  In each segment, a local trend series *y*_*v*_(*j*) is calculated with *k* order polynomial fitness. The detrended series *Y*_*s*_(*j*) is gained by subtracting the local trend from the profile. Then, the corresponding square fluctuation *F*^2^(*v*, *s*), defined as the variance of *y*_*s*_(*j*), can be captured.
  $$Y_{s}(j)=Y(j)-y_{v}(j)$$ (2)
  $$F^{2}(v,s)=\frac{1}{s}\sum_{j=1}^{s}{\left\{Y\left[(v-1)s+j\right]-y_{v}(j)\right\}}^{2},(v=1,2,\ldots ,N_{s})$$ (3)
  $$F^{2}(v,s)=\frac{1}{s}\sum_{j=1}^{s}{\left\{Y\left[N-(v-N_{s})s+j\right]-y_{v}(j)\right\}}^{2},(v=N_{s}\text{+}1,N_{s}\text{+}2,\ldots ,\text{2}N_{s})$$ (4)
Step 4. Fluctuation function.
  $$F_{q}(s)={\left\{\frac{1}{2N_{s}}\sum_{v=1}^{2N_{s}}{\left[F^{2}(v,s)\right]}^{q/2}\right\}}^{1/q},q\neq 0$$ (5)
  $$F_{\text{0}}(s)=\text{exp}\left[\frac{1}{4N_{s}}\sum_{v=1}^{2N_{s}}\text{ln}\left[F^{2}(v,s)\right]\right],q=0$$ (6)
  where, in general, the index variable *q* can take any real value. For *q* = 2, the standard DFA procedure is retrieved. With different segment lengths, log-log plots of *F*_*q*_(*s*) versus *s* can be obtained by repeating steps 2 to 4. The scaling behavior of the fluctuation functions is determined by analyzing the log-log plots. If the series {*x*_*i*_}(*i* = 1, 2, …*N*) are long-range power-law correlated, *F*_*q*_(*s*) increases. For large values of *s*, this increases as a power law.
  $$F_{q}(s)\sim s^{h(q)}$$ (7)

For a stationary time series, *h*(2) is identical to Hurst exponent *H*. Thus, we call the function *h*(*q*) the generalized Hurst exponent [6]. For positive values of *q*, the fluctuation function, *F*_*q*_(*s*), is dominated by the large variance *F*^2^(*v*, *s*), while *h*(*q*) describes the scaling behavior of segments with large fluctuations and vice versa. For mono-fractal time series characterized by a single exponent, *h*(*q*) is independent of *q*. For multi-fractal time series, *h*(*q*)varies with *q*. There is a relationship between standard multi-fractal analysis and MF-DFA. The classical multi-fractal scaling exponent *τ*(*q*) is related to *h*(*q*).

τ(q)=qh(q)−1 (8)

During standard multi-fractal analysis, the multi-fractal spectrum *f*(*α*) [32] is calculated to characterize scaling behavior. Via Legendre transform, the singularity spectrum, *f*(*α*), can be related to *τ*(*q*) [6].

$$\alpha \text{=}\tau^{\prime }(q)$$ (9)

f(α)=qα−τ(q) (10)

where α is the singularity strength, or Holder exponent, and *f*(*α*) denotes the dimension of the subset of the series characterized by α. The width Δ*α* = *α*_max_−*α*_min_ illustrates the multi-fractal degree.

### Range of segment length in WSTS scaling analysis

The segment length, *s*, is the significant parameter in confining the scope of scaling analysis. The lower bound of *s* delimits the left end of the log-log plot of *F*_*q*_(*s*) versus *s*, and thus further denotes the small temporal scale of scaling analysis. The upper bound demarcates the right end, and the large temporal scale. In Ref. [6], the range of segment lengths of MF-DFA is discussed from a mathematical perspective. It is noted that, by construction, *F*_*q*_(*s*) is only defined for s≥*k*+2. For very large scales, where *s*>*N*/4, *F*_*q*_(*s*) becomes statistically unreliable because the number of segments, *N*_*s*_, for the averaging procedure in Step 4 becomes very small. Thus, scales where *s* > *N*/4 are usually excluded from the fitting procedure when determining *h*(*q*) [6]. Generally, the range of *s* is represented as *k*+2≤*s*≤*N*/4.

During recent studies applying MF-DFA in scaling analysis of WSTS, the length of WSTS varies greatly, from as little as 1 day to as long as several months or even years. Similarly, the resolution of a WSTS might vary from 1 h to 1 d [3–4, 7–12]. With the evolution of technology, a WSTS with high resolution (e.g., 10 min or shorter) has become easier to obtain. As a result, processes occurring over a short time can be observed. For example, if the resolution is too low (e.g., >1d), the diel alternation of wind speed cannot be captured. With advances in instrumentation, WSTS’s of the same temporary length now contain more data than before, and the variation of wind speed is more accurately captured. At the same time, the calculations required for scaling analysis have increased.

**Fig 1:**
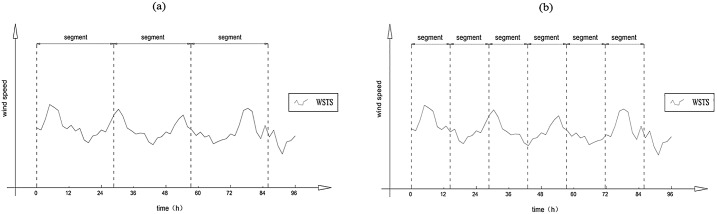
Wind speed processes.

For the lower bound of *s*, when only considering *k*, the order number of polynomial fitness might not be enough for a WSTS; when *k*≥1 and s≥*k*+2, then s≥3. This is universal for any kind of series. The parameter *s* is the data number of the segment. When multiplied by the resolution of the WSTS, it can be transferred into a temporal length. The minimum temporal length of the segment may be triple the resolution if *s*≥3. Similarly, the higher the resolution is, the shorter the minimum temporal length will be. For example, when the resolution is 10 min, the minimum temporal length is 30 min. Wind speed has the feature of diel variation with a length of nearly 24 h and is influenced by the Earth’s rotation (Fig 1). During scaling analysis of a WSTS, when the segment is longer than 1 day the diel variation can be included in a single segment. During the local trend calculation and elimination step of MF-DFA, the diel trend can be captured (Fig 1a). Otherwise, the diel variation will not be obtained and the diel trend cannot be detected (Fig 1b). The presence or absence of the diel trend in the segment induces the effect of detrending.

With the abovementioned limitation, the upper bound of *s* is *N*/4 [6]. When *N* is longer than 1 year, the upper bound of s can be larger than 1 season. Considering the influence of seasonal alternations, there may be inconsistency between super-seasonal and sub-seasonal scales; this has been examined previously [10]. The length of the WSTS used in this study is 1 year, which is not long enough to examine the issue of seasonal scale. Thus, the upper bound of *s* and the seasonal issue are not discussed in this paper.

## Results and Discussion

To determine the effect of extending the lower bound of the segment length to sub-daily, two schemes for segment length range are compared. One scheme is called long range (from 6 h to 3000 h), while the other is called short range (from 24 h to 3000 h). Based on these two schemes, the WSTS of 10 locations (Table 1) were analyzed by MF-DFA.

**Fig 2:**
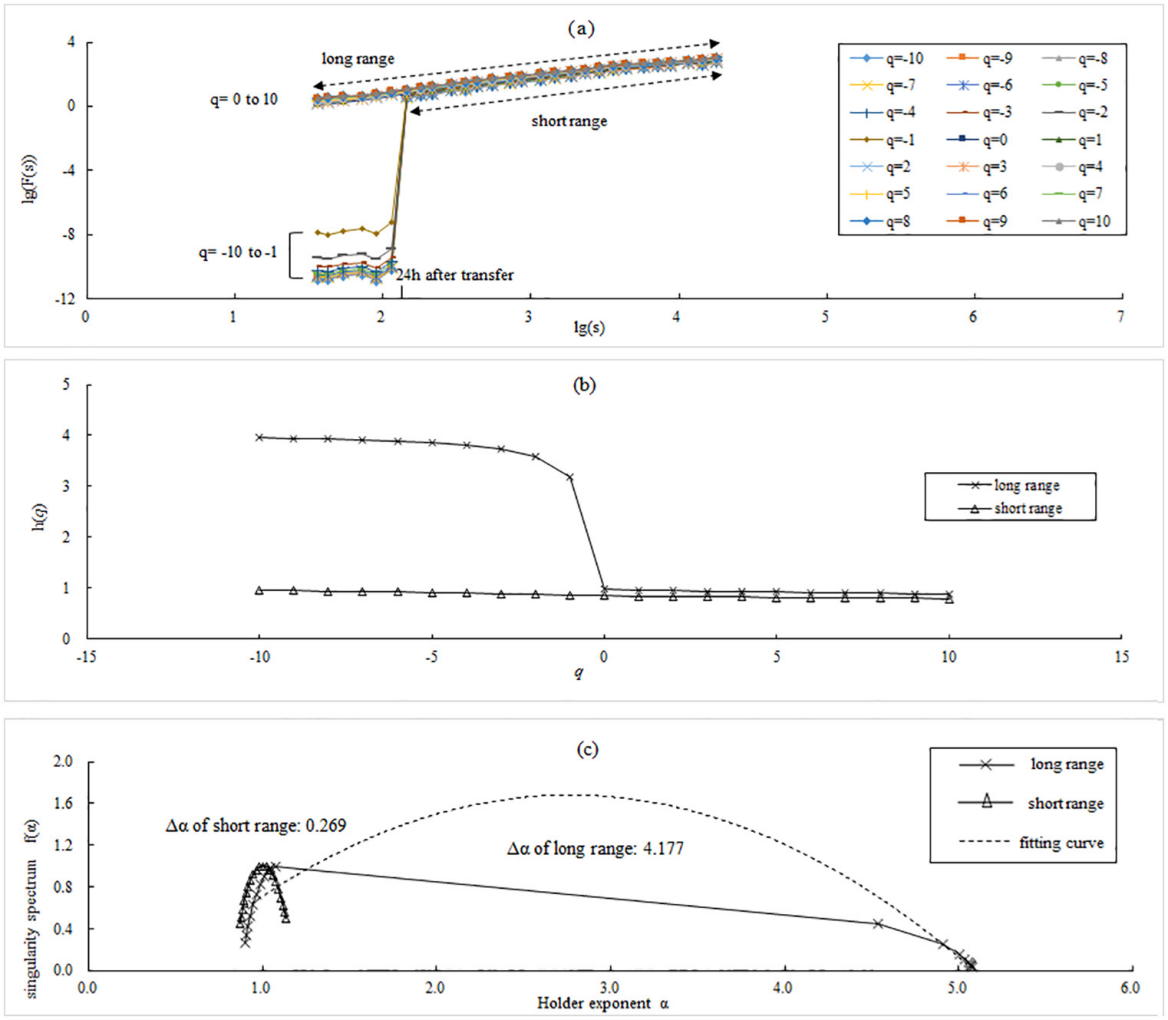
MF-DFA plots of the two schemes for 1# WSTS. (a) Log—log plots; (b) *h*(*q*)~*q* plots, and; (c) *f*(*α*) ~ *α* plots.

**Fig 3:**
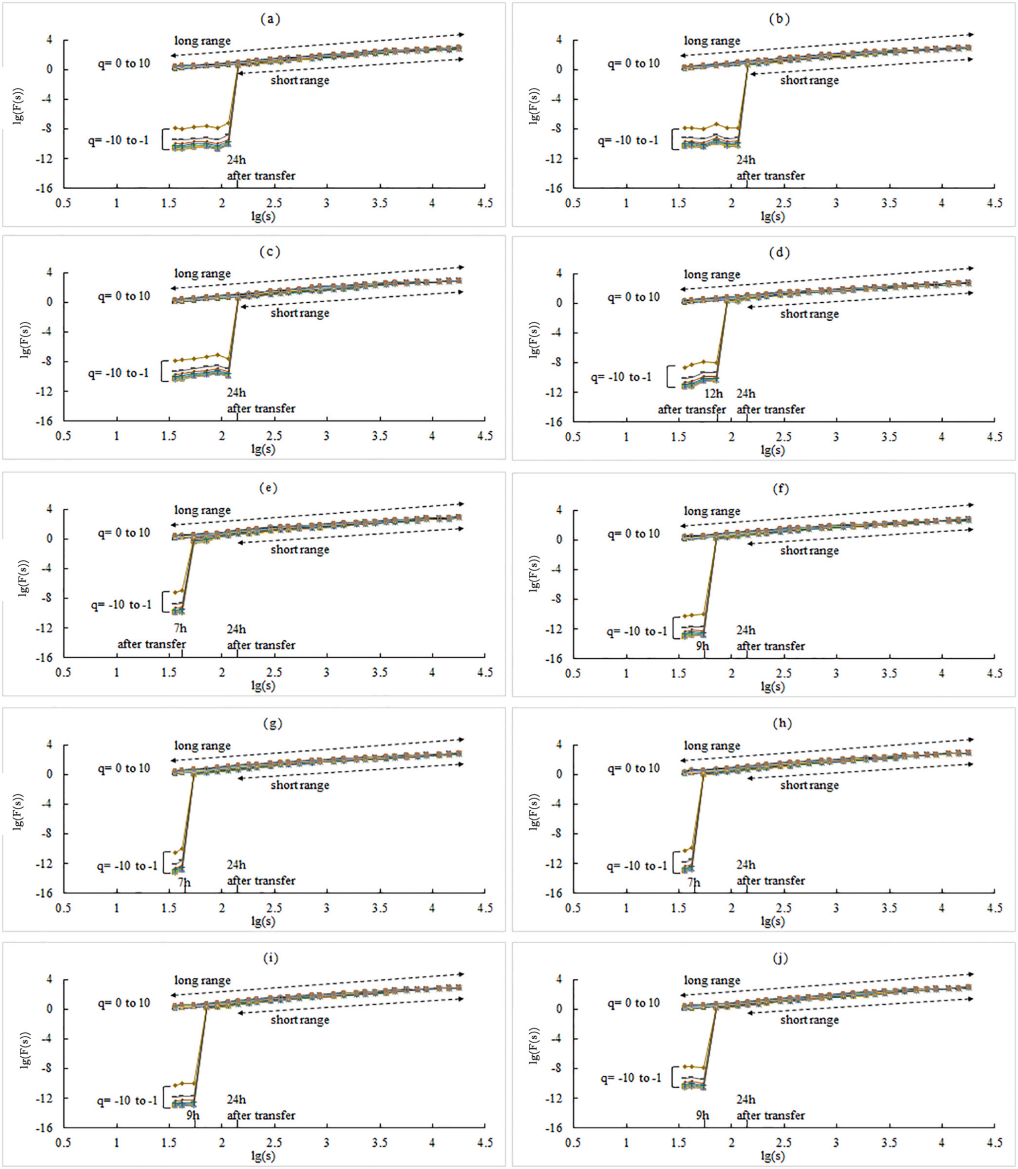
Log—log plots of WSTS. (a) plot of the 1# WSTS; (b) plot of the 2# WSTS; …, and; (j) plot of the 10# WSTS. The legend is same as in Fig 2a.

The results of 1# WSTS are shown in Fig 2. In Fig 2a, the log—log plots of the fluctuation function, *F*_*q*_(*s*), versus *s* are shown using fourth order polynomial detrending (MF-DFA4). The generalized Hurst exponent, *h*(*q*), was estimated with corresponding *q*, which varies from -10 to 10. The plots of *h*(*q*) ~ *q* for the two schemes are shown in Fig 2b. The plots of *f*(*α*) ~ *α* for the two schemes are displayed in Fig 2c. Extending the range of *s* induces differences (Fig 2c). In the log—log plots, there is an abrupt decline at the left part where the segment length, *s*, is less than 24 h; this decline also occurs in the other WSTS (Fig 3). The time nodes of the declines are also different, ranging from 7 h to 19 h (Fig 3). This may be related to the sub-daily feature of local meteo-climatic phenomena. For the *h*(*q*)~*q* plots of 1# WSTS, when *q* is smaller than 0, *h*(*q*) shows a notable increase in the long range scheme. Similar results are also found in the other WSTS’s (data not shown). The Holder exponent (Δ*α*), an important parameter of scaling analysis, has abnormalities in the long range scheme (Fig 2c). In the short range scheme, plots of the Holder exponent are similar to prior works. If *s* is extended into the sub-daily, the discrepancy value of Δ*α* increases from 1.492 to 3.908, and its percentage increases from 430.2% to 1455.6% (Table 2).

**Table 2:** Holder exponents of various WSTS in long/short range scheme.

Site No.	Δ*α*, Holder exponent		Discrepancy	
	Long range	Short range	Value(Long range minus short range)	Percentage (%)
**1#**	4.177	0.269	3.908	1455.6
**2#**	4.087	0.353	3.734	1057.4
**3#**	4.041	0.390	3.650	935.2
**4#**	3.226	0.273	2.952	1079.9
**5#**	1.813	0.321	1.492	465.1
**6#**	3.059	0.331	2.729	825.3
**7#**	2.244	0.360	1.884	523.0
**8#**	2.265	0.427	1.838	430.2
**9#**	3.088	0.358	2.730	763.1
**10#**	2.541	0.355	2.186	615.0

When the segment length, *s*, is shorter than 1 day, the diel variation cannot be included in one segment (Fig 1a). To determine if the diel variation is the cause of the observed differences, a shuffled disposal was adopted. To distinguish between the two, the un-shuffled WSTS is called the chronological WSTS. There were two shuffled WSTS. The first was shuffled according to date and is called the inter-day shuffled WSTS, which means that the diel variation is maintained and there is no intra-day switch. The second was shuffled within the scope of each single day and is called the intra-day shuffled WSTS, which means that there is no exchange between two different days. Each shuffled WSTS was analyzed by the foregoing two schemes of *s* (short range and long range).

**Fig 4:**
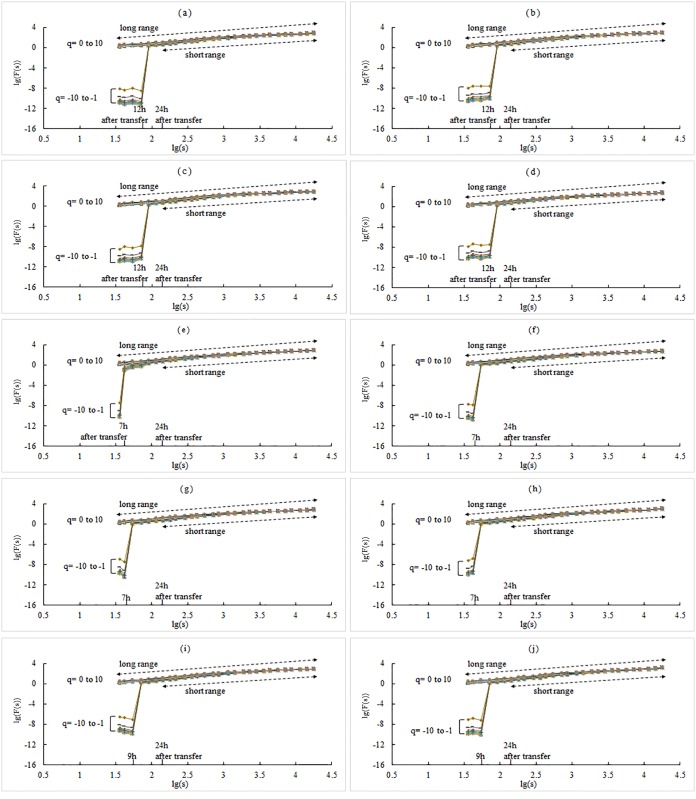
Log—log plots of the inter-day shuffled WSTS. (a) plot of the 1# WSTS; (b) plot of the 2# WSTS; …, and; (j) plot of the 10# WSTS. The legend is same as in Fig 2a.

**Fig 5:**
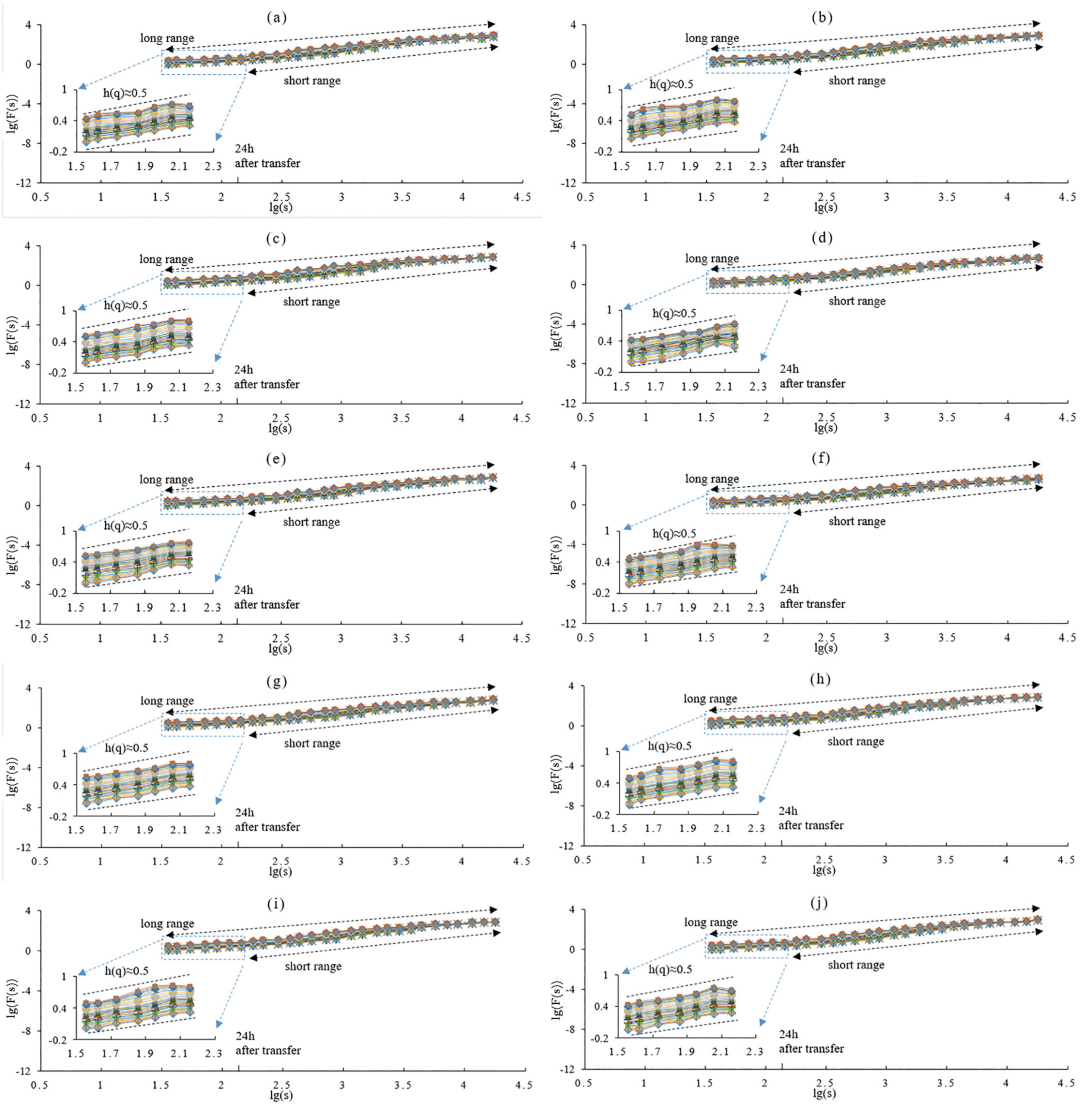
Log—log plots of the intra-day shuffled WSTS. (a) plot of the 1# WSTS; (b) plot of the 2# WSTS; …, and; (j) plot of the 10# WSTS. The legend is same as in Fig 2a.

The results of the inter-day shuffled WSTS show that the abrupt declinations still exist in the long range scheme (Fig 4). However, in the intra-day shuffled WSTS, there is no sign of abrupt declination in the left part of plots (Fig 5). Furthermore, in the sub-daily scope of *s*, the local *h*(*q*) is nearly 0.5. That states its intra-day process of WSTS is random, which coincides the effect of intra-day shuffled. Comparison of the inter-day shuffled WSTS and the intra-day shuffled WSTS revealed that if the diel variation is retained and *s* is extended into sub-daily (i.e., the long range scheme in Fig 4), there will be abrupt declinations that induce discrepancies in scaling exponents; however, without diel variation no such phenomena emerge (i.e., long range scheme in Fig 5). These results illustrate that the diel variation is the reason for the declination and associated discrepancies. This result supports our initial assumptions. The next logical question is if diel variation can be ignored. For the short range scheme of the intra-day shuffled WSTS, where the diel variation is removed, the Holder exponents are listed in Table 3. Compared with the short range scheme of chronological WSTS, we found a shift in the scaling exponent from -0.103 to 0.074 (-22.4% to 25.9%, respectively). Therefore, removal of diel variation is not appropriate. We, therefore, propose that the diel variation should be kept and the lower range of *s* should be larger than 1 day to avoid discrepancies in scaling analysis.

**Table 3:** Holder exponents of chronological WSTS and intra-day shuffled WSTS in short range scheme.

Site No.	Δ*α*, Holder exponent		Discrepancy	
	Chronological	Intra-day shuffled	Value(Chronological minus intra-day shuffled)	Percentage (%)
**1#**	0.269	0.326	-0.057	-17.6
**2#**	0.353	0.335	0.018	5.5
**3#**	0.390	0.481	-0.090	-18.8
**4#**	0.273	0.232	0.042	18.0
**5#**	0.321	0.324	-0.003	-1.0
**6#**	0.331	0.355	-0.025	-6.9
**7#**	0.360	0.286	0.074	25.9
**8#**	0.427	0.435	-0.007	-1.7
**9#**	0.358	0.412	-0.054	-13.2
**10#**	0.355	0.458	-0.103	-22.4

**Fig 6:**
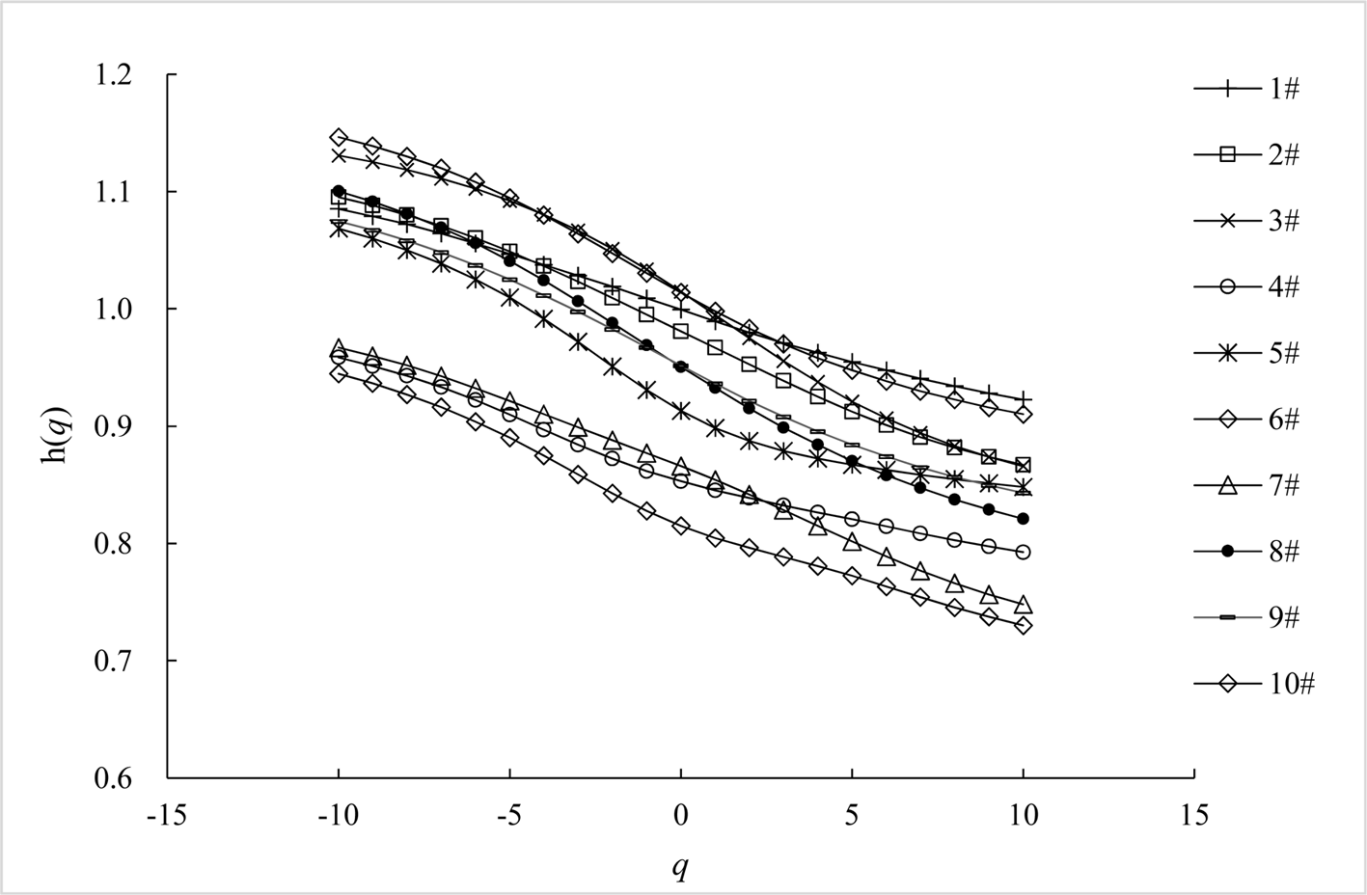
The *h*(*q*) ~ *q* plots of the short range scheme of our WSTSs.

**Fig 7:**
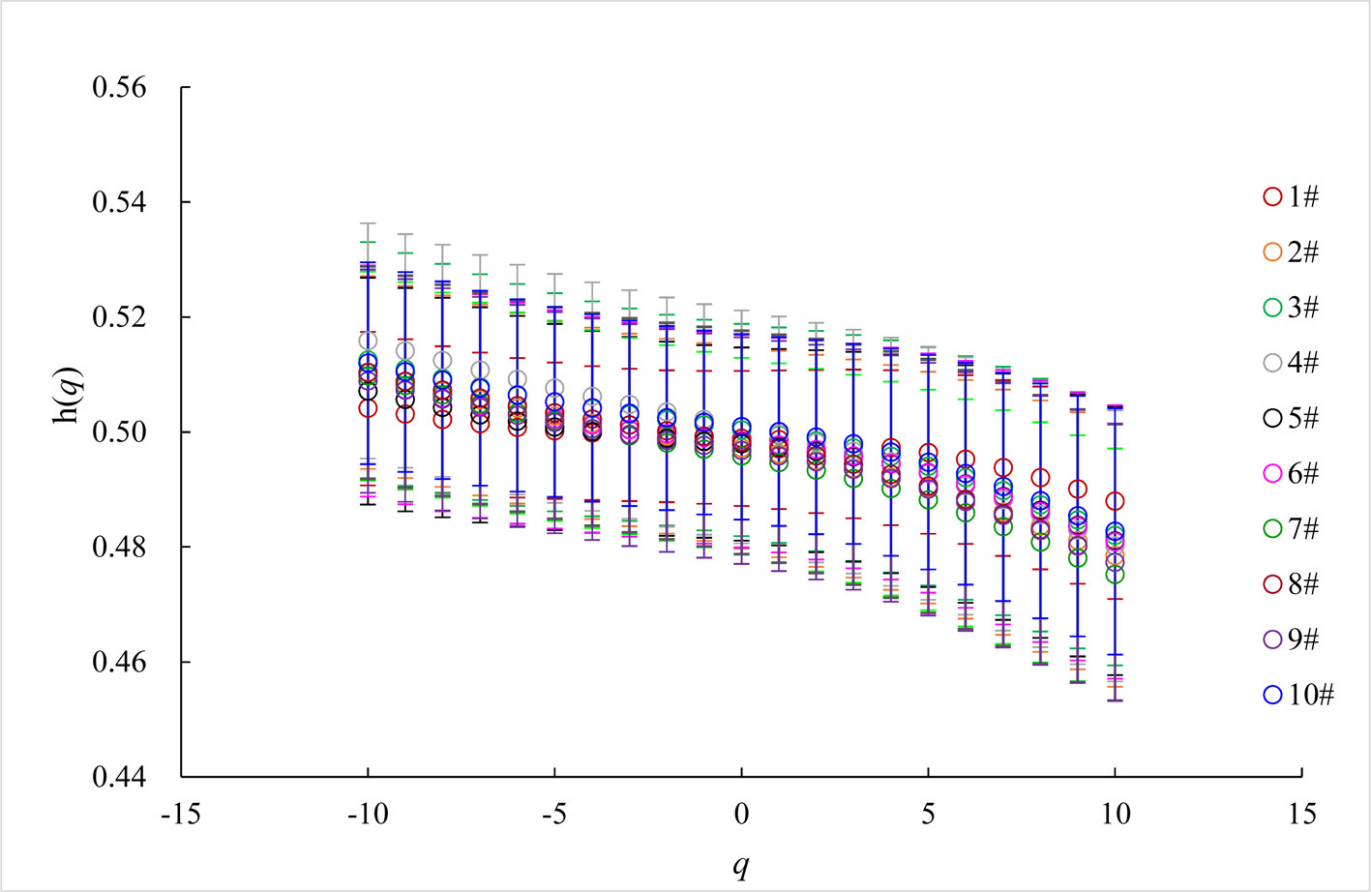
The *h*(*q*) ~ *q* plots of 100 surrogate series for each WSTS (mean and error bar).

The short range scheme of the chronological WSTS we analyzed meets the requirements of diel variation and the lower range of *s*. For our WSTSs, *h*(*q*) is a nonlinear function of *q* (Fig 6); this is a hallmark of multi-fractality [33–34]. Multi-fractality of a time series can be due to: (i) a broad probability density function for the values of the time series, or; (ii) different long-range correlations for small and large fluctuations [6]. To distinguish the specific type of multi-fractality for our WSTSs, we applied a fully shuffle method to generate 100 surrogate series for each WSTS. The shuffle was for the entire scope of each time series, as opposed to being either inter-day or intra-day. Fig 7 shows the *h*(*q*) ~ *q* plots of 100 surrogate series for each WSTS averaged over 100 surrogate series. The error bars demarcate the 1-σ range around the mean values. The mean h(*q*) values have a range of approximately 0.5 for all WSTS, with a slight *q*-dependence (Fig 7). This illustrates that the multi-fractality of these WSTSs is due to different long-range correlations for small and large fluctuations. This result agrees with previous work on the multi-fractality of wind.

## Conclusion

We have discussed the influence of diel variation on MF-DFA of WSTS from the perspective of a lower range of segment length (*s*) and the intra-day order of wind. The range of *s* during general scaling analysis was introduced. Considering the improvements in anemometer equipment and the diel variation feature of wind speed, the proper lower bound of *s* was pursued. Extending the lower bound of *s* into the sub-daily results in an abrupt decline and discrepancies in scaling exponents. We propose that the lower range of *s* should be longer than 1 day to avoid these discrepancies. Comparison of WSTSs with and without diel processes showed pronounced differences. Therefore, the intra-day order of WSTS should not be ignored during scaling analysis.

During scaling analysis of WSTSs, the natural feature of wind, diel variation, should be accounted for. However, there are also other features, such as seasonal alternation, local meteorological patterns, and other natural laws, which may impact the wind; these would be analyzed in future works.

## Supporting Information

S1 Fig Process of 1# WSTS. (TIF)

S2 Fig Process of 2# WSTS. (TIF)

S3 Fig Process of 3# WSTS. (TIF)

S4 Fig Process of 4# WSTS. (TIF)

S5 Fig Process of 5# WSTS. (TIF)

S6 Fig Process of 6# WSTS. (TIF)

S7 Fig Process of 7# WSTS. (TIF)

S8 Fig Process of 8# WSTS. (TIF)

S9 Fig Process of 9# WSTS. (TIF)

S10 Fig Process of 10# WSTS. (TIF)
