# Supplementary figures and images for: Influence of Sub-Daily Variation on Multi-Fractal Detrended Fluctuation Analysis of Wind Speed Time Series
Source: PLoS One. 2016 Jan 7;11(1):e0146284. doi: 10.1371/journal.pone.0146284 (PMC4711791; doi:10.1371/journal.pone.0146284)

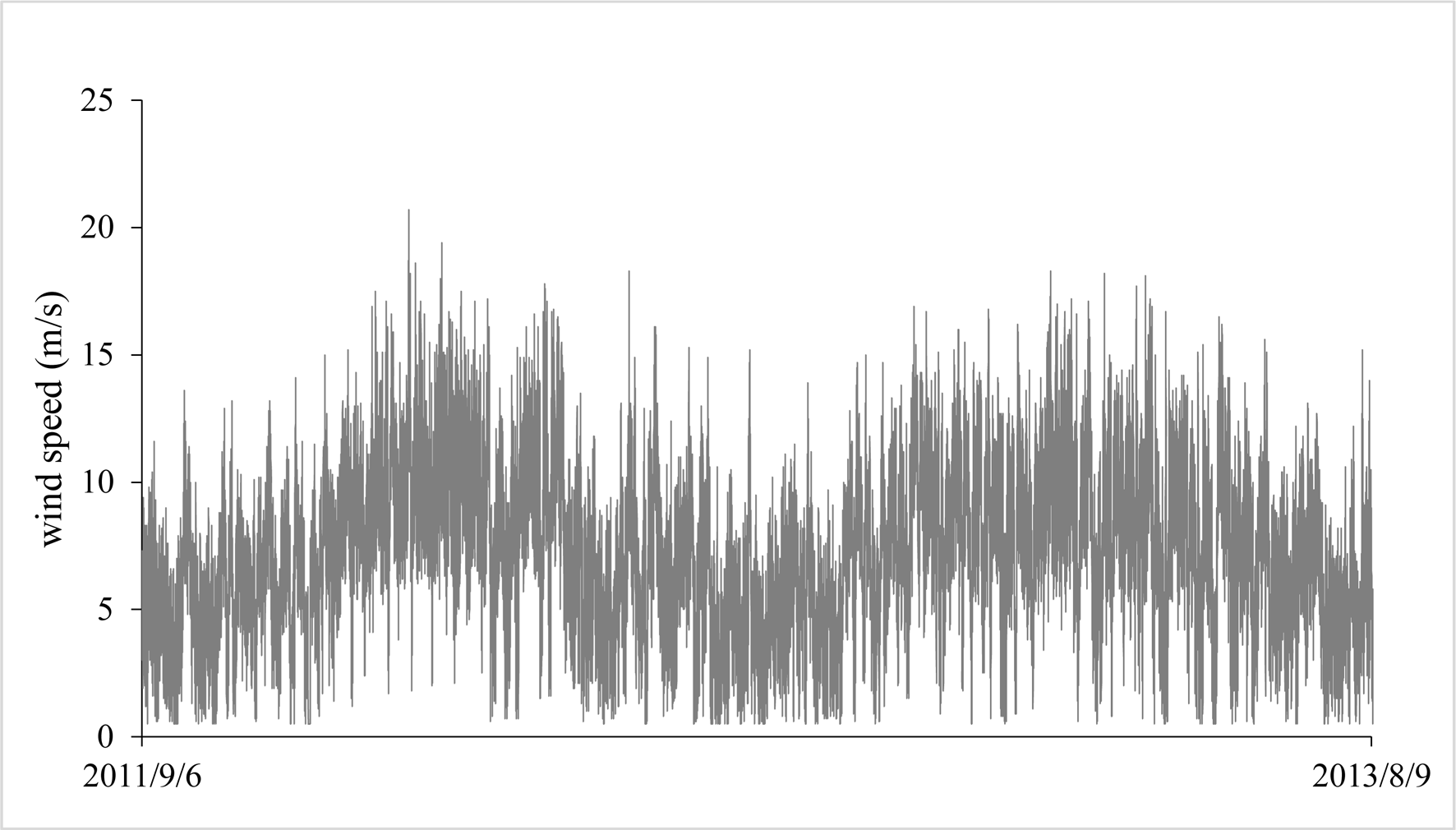

Supplement: S1 Fig — (TIF) [file pone.0146284.s001.tif]

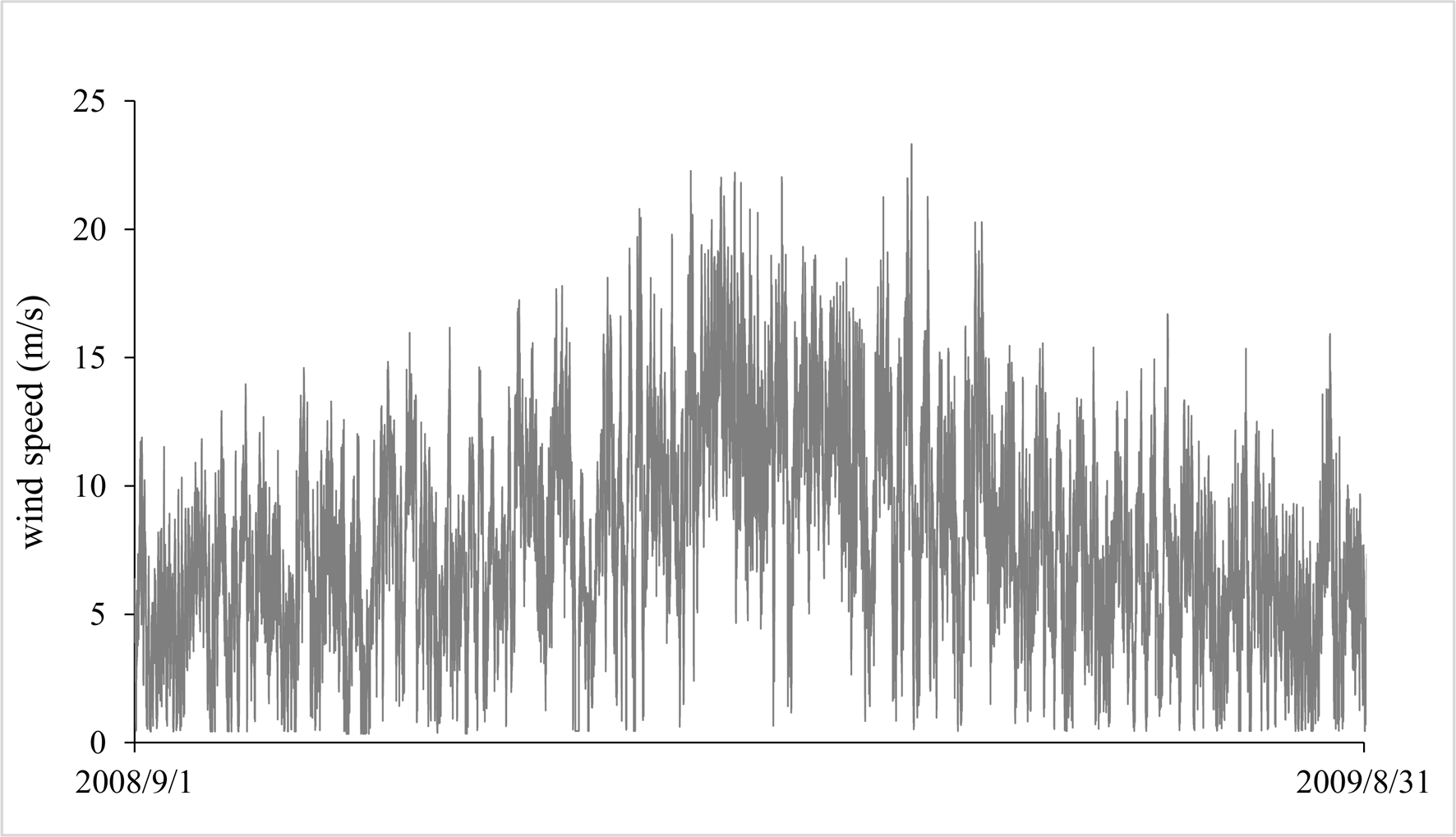

Supplement: S2 Fig — (TIF) [file pone.0146284.s002.tif]

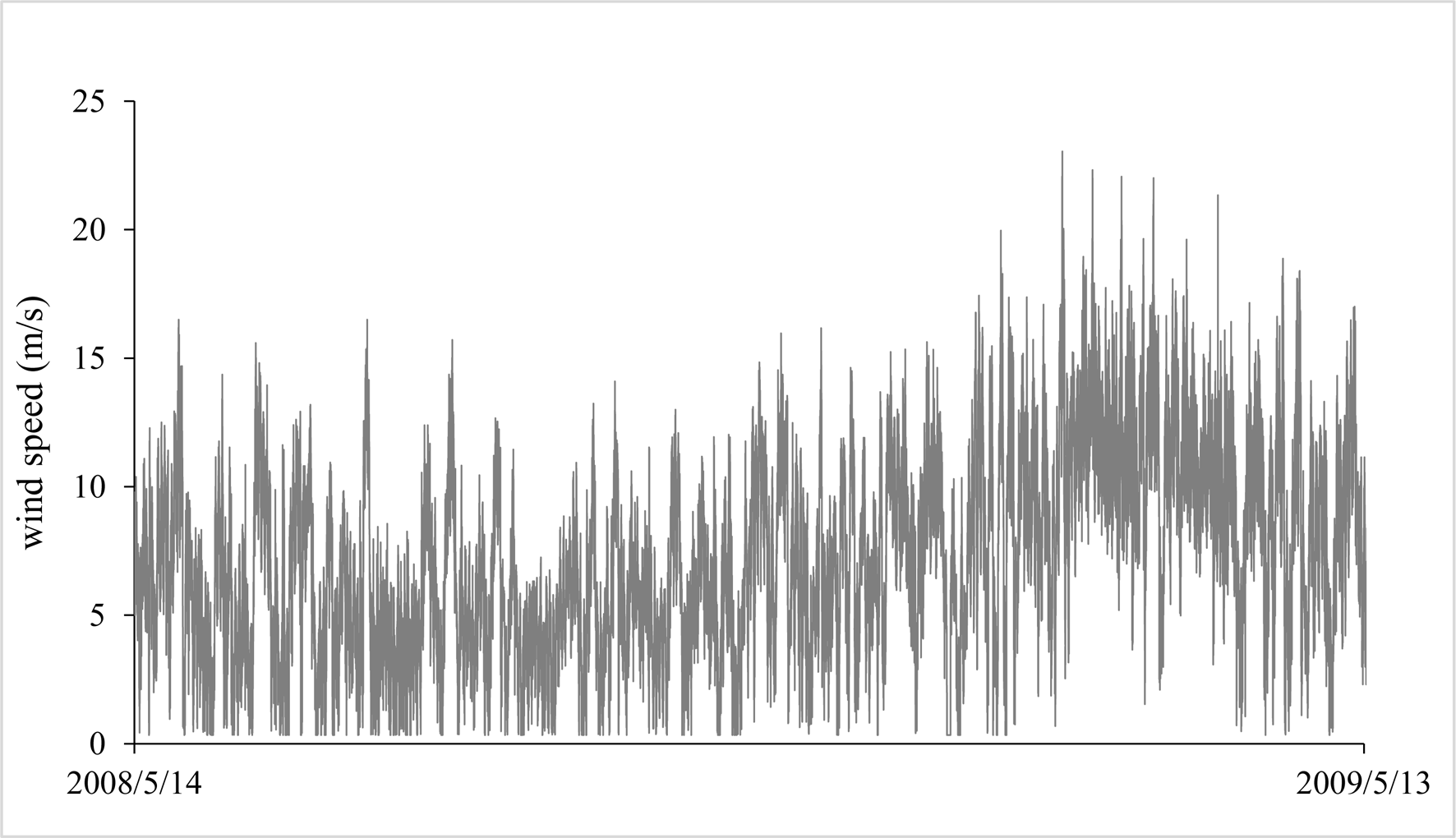

Supplement: S3 Fig — (TIF) [file pone.0146284.s003.tif]

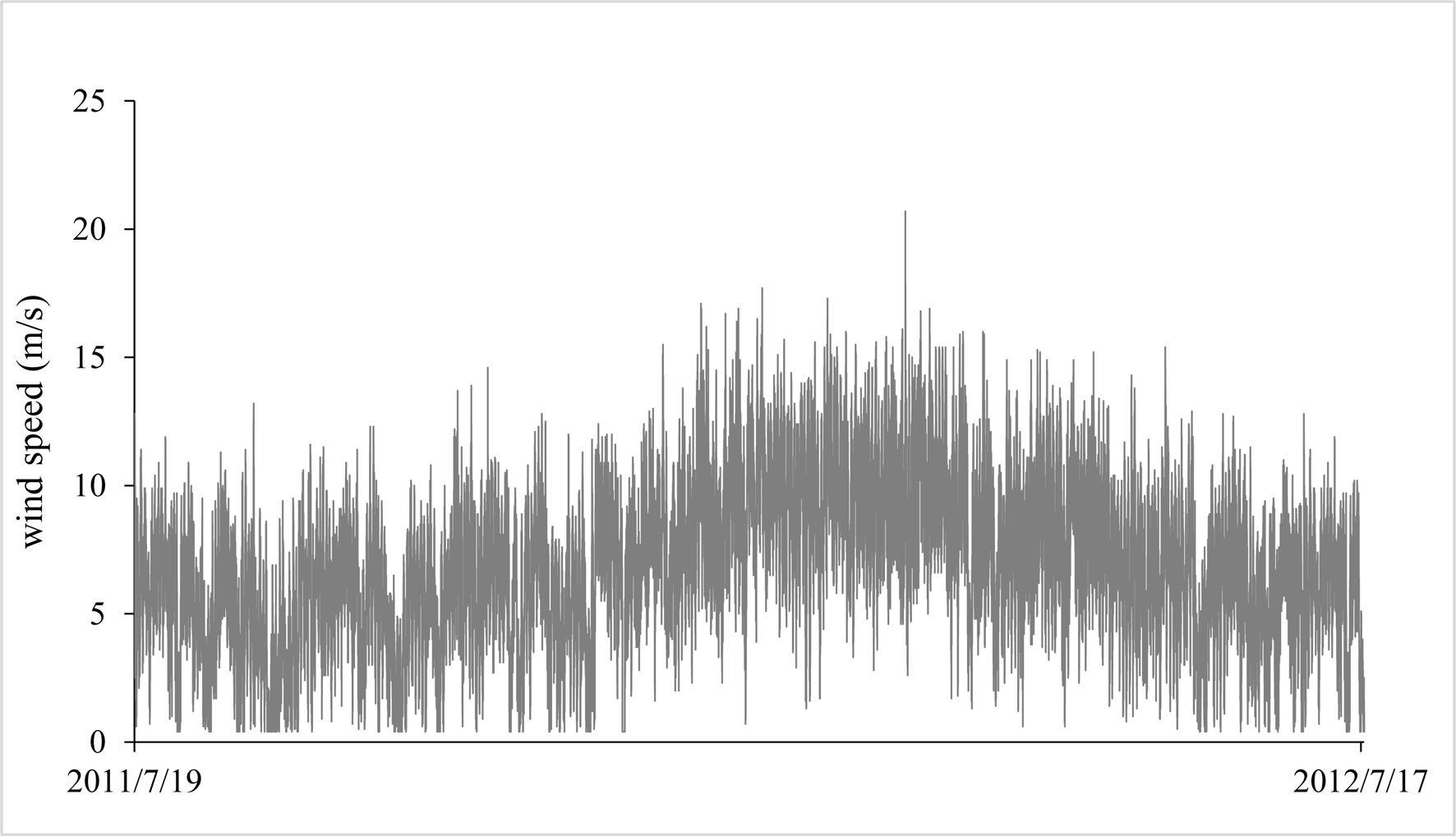

Supplement: S4 Fig — (TIF) [file pone.0146284.s004.tif]

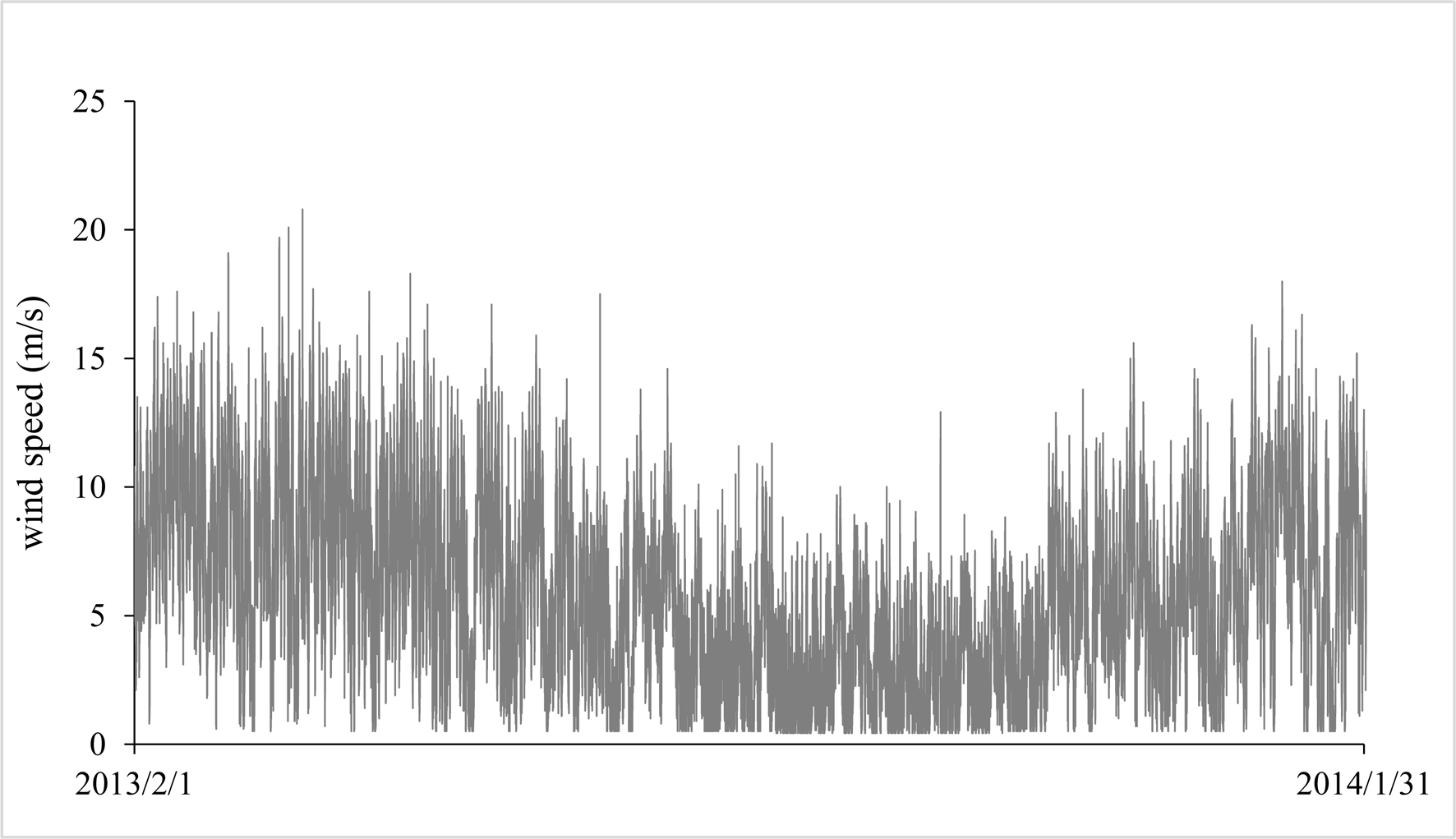

Supplement: S5 Fig — (TIF) [file pone.0146284.s005.tif]

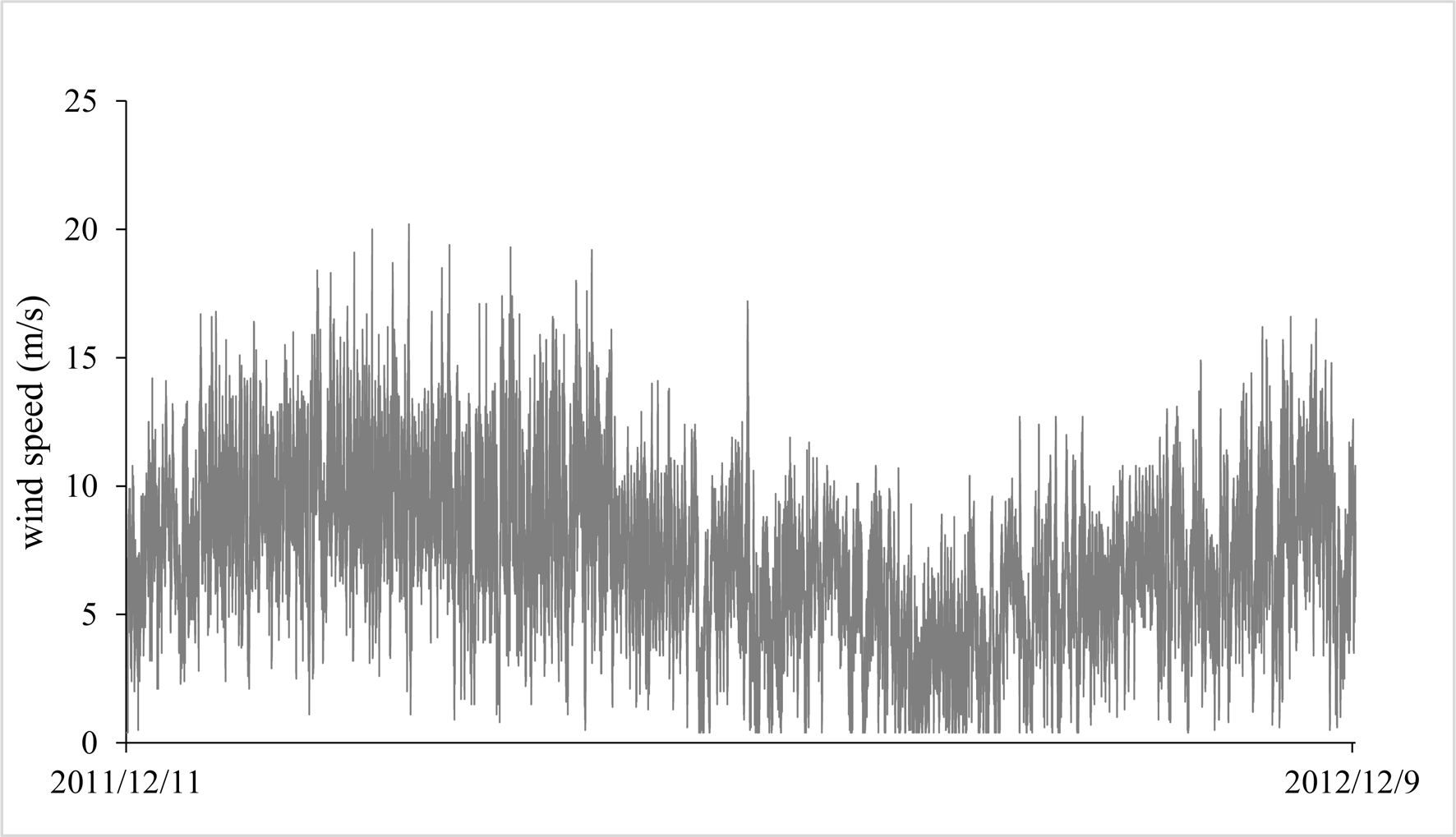

Supplement: S6 Fig — (TIF) [file pone.0146284.s006.tif]

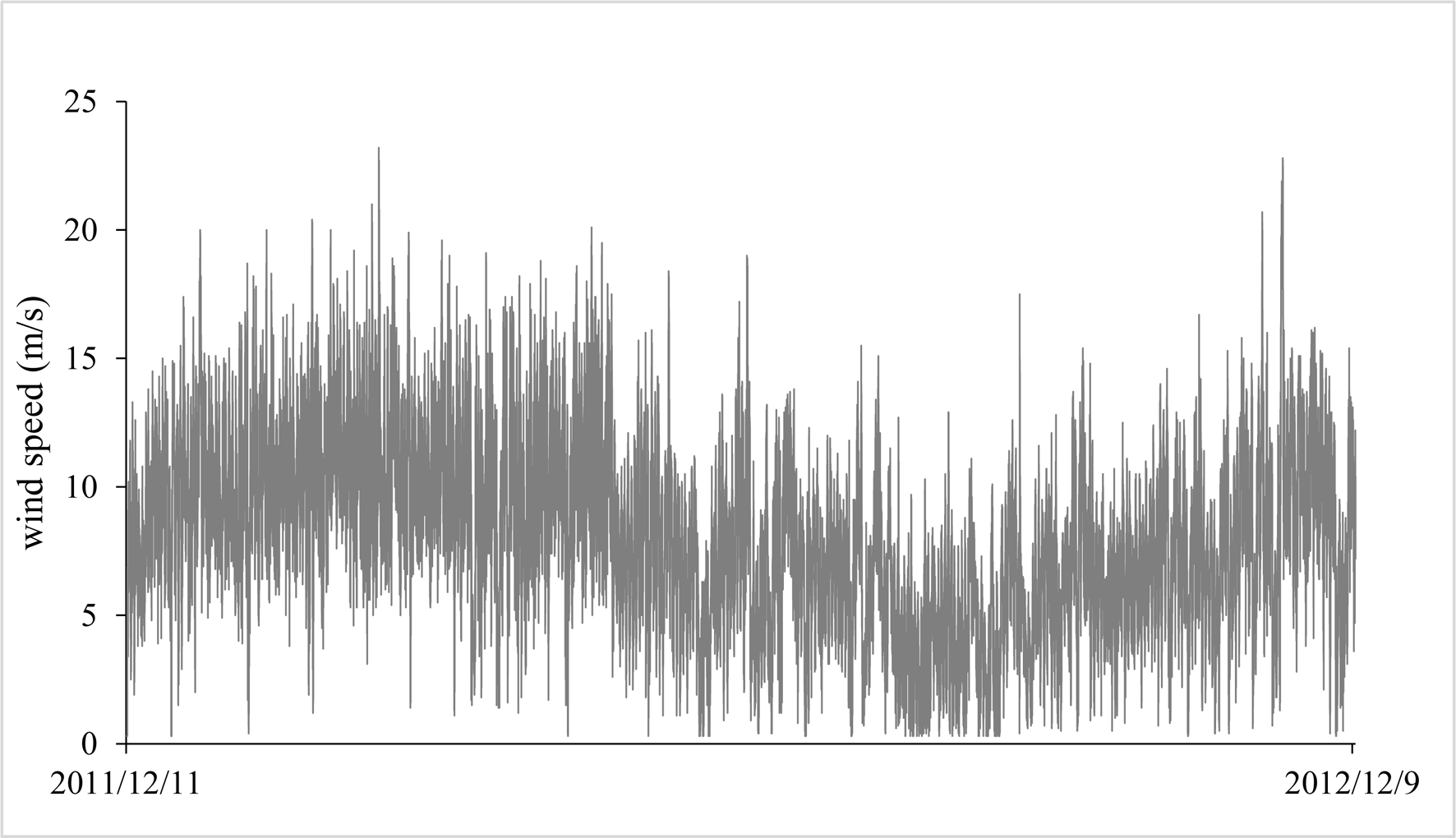

Supplement: S7 Fig — (TIF) [file pone.0146284.s007.tif]

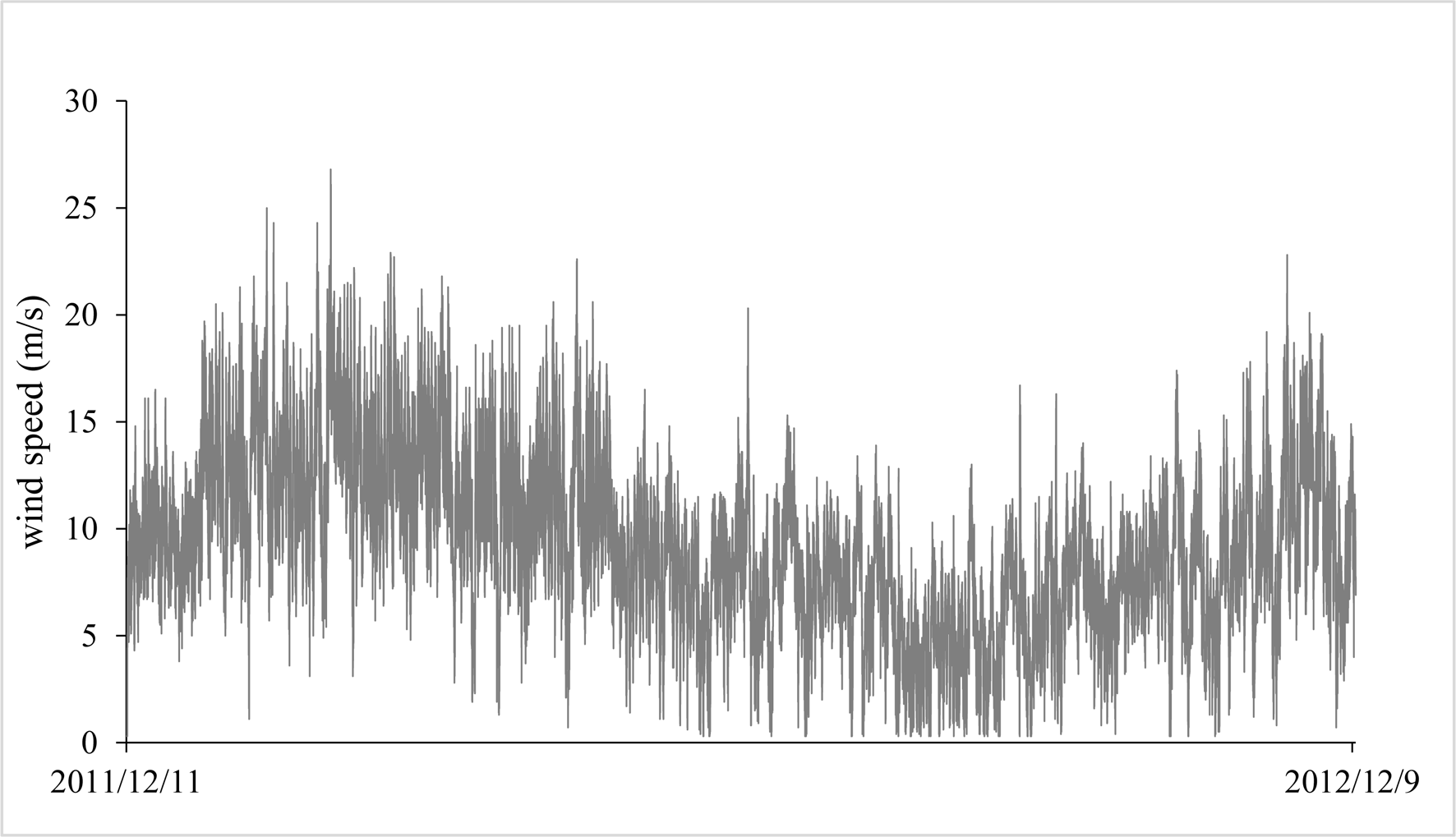

Supplement: S8 Fig — (TIF) [file pone.0146284.s008.tif]

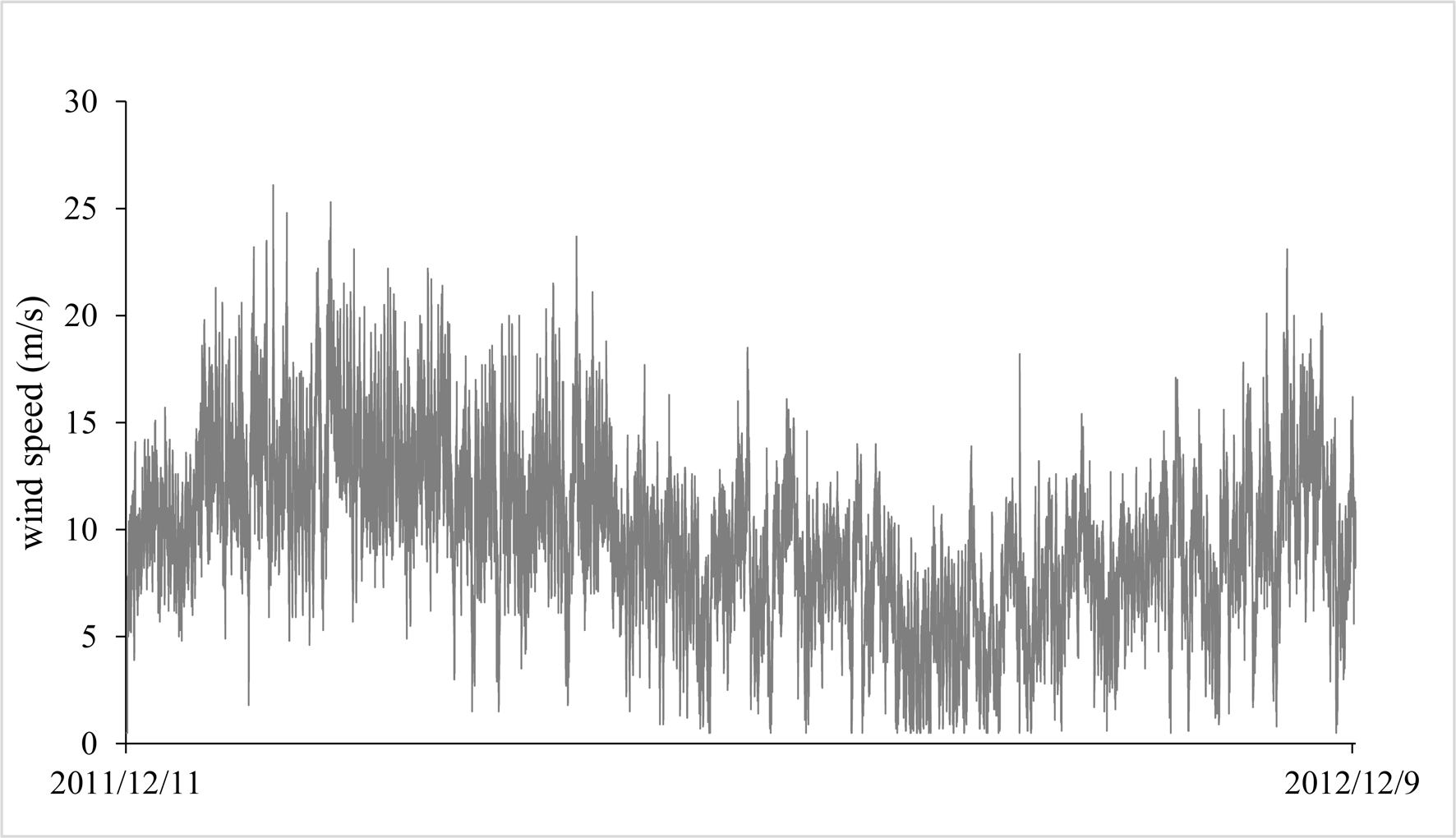

Supplement: S9 Fig — (TIF) [file pone.0146284.s009.tif]

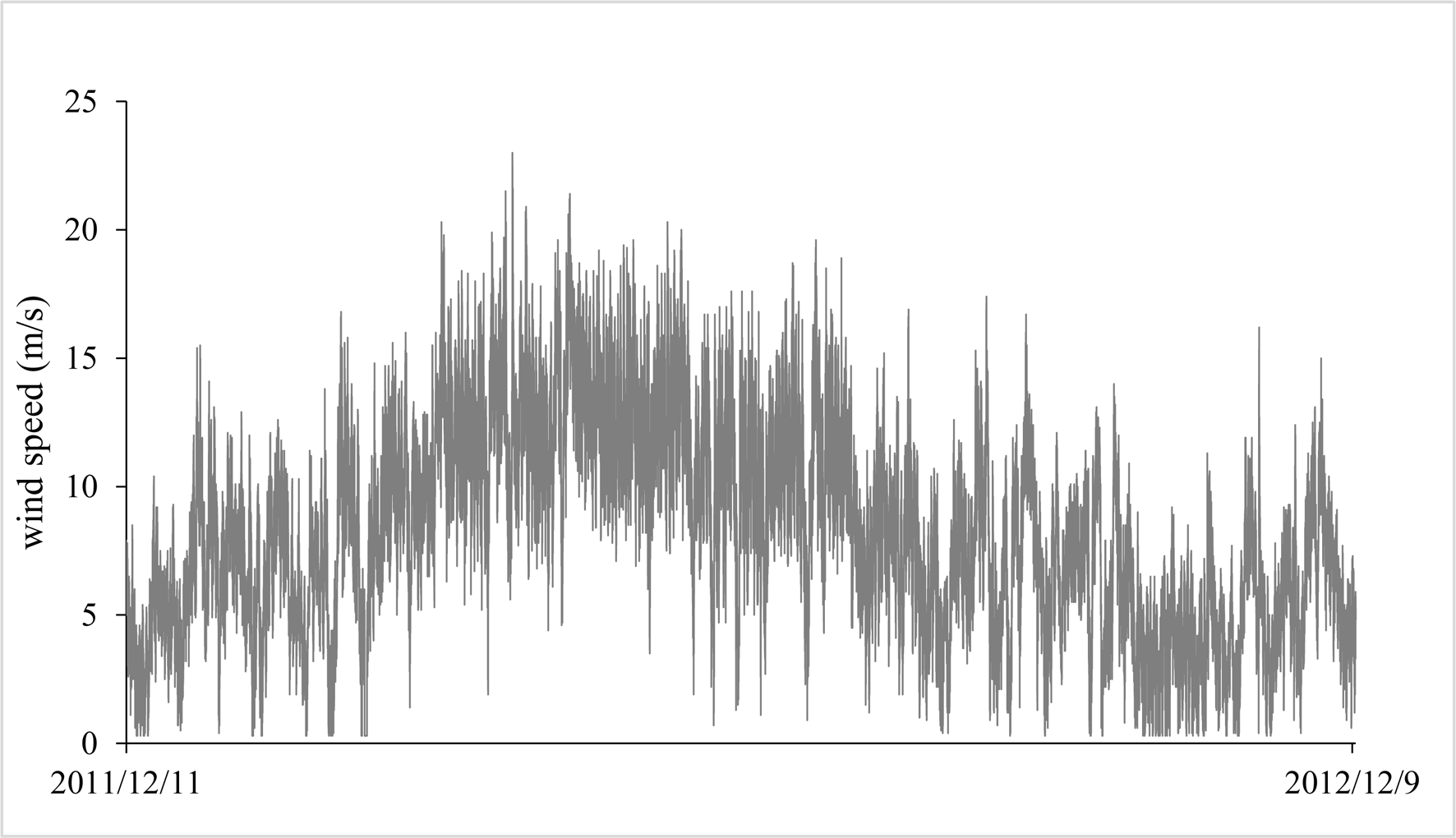

Supplement: S10 Fig — (TIF) [file pone.0146284.s010.tif]
